# System-Wide Implementation of Colorectal Cancer Screening in a Value-Based Care Setting

**DOI:** 10.1007/s11606-025-09706-0

**Published:** 2025-07-14

**Authors:** Kimon Stathakos, John Hon, Lindsey Palazzo, Doran Kim, Anne Flynn, Juan Carlos Bucobo, Zenobia Brown, Eun Ji Kim

**Affiliations:** Northwell, New Hyde Park, NY USA

**Keywords:** Colorectal cancer screening, Value-based care, FIT, Implementation, Quality improvement

## Abstract

**Background:**

Colorectal cancer (CRC) screening is a HEDIS measure in value-based care (VBC), but the screening rate among patients in VBC is suboptimal.

**Objective:**

To increase CRC screening through home-based fecal immunochemical test (FIT) among patients in VBC.

**Design:**

Observational study.

**Participants:**

We included patients aged 45–75 years in VBC (4 Medicare, 1 Medicaid plan) attributed to Northwell Health’s provider panels who had not completed CRC screening for 2023 in October 2023.

**Intervention:**

The primary exposure is mailed FITs to patients’ homes from November to December 2023. Patients who had not completed the kits were reached through a series of three telephone calls 3 weeks after kits were delivered. For patients with abnormal results, we coordinated fast-track referrals to gastroenterology or colonoscopy.

**Main Measures:**

The primary outcome of interest is the number and proportion of completed FIT kits. Our secondary outcome of interest is the Centers for Medicare & Medicaid Services STAR Quality Rating for each corresponding VBC plan.

**Key Results:**

Out of 3680 kits mailed, 3466 (94.2%) kits were delivered. Among kits delivered, 465 (13.4%) kits were completed. We found that patients who had an appointment with providers within the last 18 months had a higher completion rate (15.9%) compared to patients who did not have a visit or had a visit more than 18 months ago (9.3%) (*p*-value < 0.0001). Among 45 patients with abnormal results (9.7%), 11 patients (24.4%) completed diagnostic colonoscopies and 10 patients (90.0%) were found to have tubular adenomas (May 2024). This initiative resulted in a 1 STAR increase across four value based care programs (2 Medicaid, 2 Medicare).

**Conclusions:**

The population health initiative at scale to increase CRC screening resulted in a small, but meaningful improvement. There remain opportunities to improve CRC screening and treatment by coordinating diagnostic colonoscopies for this population.

**Supplementary Information:**

The online version contains supplementary material available at 10.1007/s11606-025-09706-0.

## INTRODUCTION

Colorectal cancer (CRC) is the second highest cancer-related death and third highest diagnosed cancer in the United States (U.S.).^1^ The guideline by the United States Preventive Services Task Force (USPSTF) was expanded to start at age 45 instead of 50 as a result of the substantial increase in the incidence of CRC in adults 40–49.^2^ The CRC screening rate in the U.S. remains suboptimal despite being a quality measure used to evaluate health systems by both commercial and governmental insurers.^3^ While colonoscopy is a first-line recommendation for CRC screening, home-based stool testing has been shown to reduce the mortality rate from CRC.^4^ The home-based stool testing completion rate has also been demonstrated to be higher than colonoscopy.^5^ Prior studies have shown that fecal immunochemical test (FIT) initiatives have high rates of success in CRC screening as well as cost-effectiveness.^6,7^ Screening tests are inherently beneficial on a population level, and rates of screening are an established means in which health systems are evaluated and financially incentivized by payer organizations.

There are different interventions to support CRC screening through FIT-based screening programs.^8^ The use of mail-in kits with follow-up reminders through a variety of modalities (phone, in-person, etc.) helped increase FIT completion rates.^9^ An additional use of patient navigators, as members of a multidisciplinary CRC screening program, improved CRC screening rates. ^10^ CRC screening through FIT screening programs demonstrated higher participation, but it is unclear whether there was follow-up to ensure participants with a positive FIT received diagnostic colonoscopies.^11,12^ Furthermore, few studies have shared the effectiveness of integrated and multidisciplinary design.^13^ Many of these studies were conducted independently of payer incentives and other system-level strategies to modify healthcare practices. While individual studies may be able to demonstrate localized evidence to show efficacy in a limited context, a population health approach that leverages payer incentives may shed more light on the sustainability of these programs in the long term.

Value-based care (VBC) programs are incentive payments from the Centers for Medicare & Medicaid Services (CMS) based on improvements in STAR ratings and other metrics.^14^ Payers use Healthcare Effectiveness Data and Information Set (HEDIS) measures, a set of standardized performance metrics which include cancer screening, medication adherence, and other quality metrics. The goal of VBC programs is to achieve improvements in HEDIS quality measures by meeting STAR rating quality targets set forth by CMS and the National Committee for Quality Assurance (NCQA). STAR ratings are based on different thresholds needed to achieve a minimum of 1 star (poor performance) and up to 5 stars (excellent performance). With a VBC approach, a greater emphasis is placed on preventative screenings, which are measured by STAR ratings and also translate to financial incentives. The goal of assessing preventive measures, such as CRC screening, would be to identify CRC earlier and benefit from both the avoidance of greater healthcare utilization and better patient outcomes. VBC programs are increasingly being adopted as a means for the government to shift from fee-for-service to more longitudinal quality-based compensation models while controlling costs with payers. In addition to its broader adoption, policymakers have continued to advance regulatory incentives for VBC programs across health systems.^15^

Health systems have used a combination of manual and automated technologies as well as a variety of communication methods to engage patients in screening.^16,17^ At our health system, we have tried office-based outreach, mostly human-driven and a few automated, and clinics were responsible for managing patient outreach (by phone or email). The manual office process resulted in a 7% kit completion rate (200 completed kits per year) at our health system. We identified three potential intervention areas to increase kit completion by centralizing the ordering and follow-up process: increasing the number of FIT orders placed in the electronic health record (EHR), deploying and tracking kits, and ensuring patient follow-up to provide education and reminders—all within a shorter timeframe to meet VBC program deadlines (December 31st each year).

This study seeks to contribute to the growing evidence covering effectiveness, scalable volume, cost, quality metrics, and the entire CRC screening process. For this initiative, we operationalized at-scale bulk order placement and kit mailing and ensured appropriate follow-ups for positive FITs. While a diversity of CRC screening programs exist, limited literature shows how CRC screening programs, utilizing a population health approach, can affect performance in VBC models. Furthermore, while many CRC screening programs focus on improving screening rates, not many programs also include downstream efforts to ensure patients obtain diagnostic colonoscopies. VBC programs have received increasing amounts of public interest as a means in which to address a looming healthcare cost crisis. This paper examines the implementation of CRC screening initiatives in VBC models and how it impacts quality ratings and financial incentives. We are also interested in examining the end-to-end workflow from patient identification, outreach, follow-up for screening, result review, and subsequent action for abnormal results for additional patient engagement and gastroenterology services.

## METHODS

### Study Population

Northwell Health operates 21 hospitals and 900 outpatient facilities across Long Island, Westchester, and the New York metropolitan area. The health system participates in VBC contracts with multiple payers from national and regional health plans that manage over 90,000 VBC members. For this initiative, we included patients from (1) four plans that included both managed Medicaid and Medicare Advantage members, (2) had existing records in the EHR so that the results could be uploaded and communicated with their providers, and (3) had not completed a CRC screening (colonoscopy, sigmoidoscopy, FIT-DNA, or FIT), identified to be non-compliant by the payer. We used the payers’ data to identify patients who had not completed a CRC screening, confirmed through the health system’s EHR, and performed manual chart reviews.

### Colorectal FIT Program

The gaps in care (GIC) team, composed of multiple stakeholders in the health system to manage patients’ gaps in care, used the HEDIS definition to identify patients eligible for CRC screening.^18^ Patients are eligible for their CRC screening if they are between 45 and 75 years old and have not completed a colonoscopy in the past 10 years, CT colonography or flexible sigmoidoscopy in the past 5 years, FIT-DNA (e.g., Cologuard) in the past 3 years, or fecal occult blood test or FIT in the past year. This curated list was used to place orders and deploy 3680 FIT kits to patients assigned to 590 primary care providers (PCP) in 212 offices. For this program, we used FIT kits from Polymedco (specificity 94.9% and sensitivity 73.8–84.5%).^19^ Then, we created a bulk patient list and placed FIT orders under the medical director of the care management department with the Lab team. The Lab team then generated an order file (with patient demographics and specimen details). The GIC team transmitted this securely to the FIT vendor which created labels (with bar codes for the Lab team to be able to read, process, and return results directly to the EHR). The vendor sent pre-kit letters (Appendix) 3 days before kits were mailed, to help engage the patient. The letter included information about CRC to educate patients on the prevalence and survival rate, different screening options, what to expect when the kit arrived, and what to do if they had questions. A second letter was included with each kit when it was sent to reiterate the importance of CRC screening, along with an English and Spanish instruction sheet. The kit contained patient labels (name, date of birth, and collection date) and pre-addressed envelopes for convenience. Kit delivery was tracked using USPS mailing, and three interactive voice response (IVR) automated calls were made to remind patients of their screenings.

**Figure 1:**
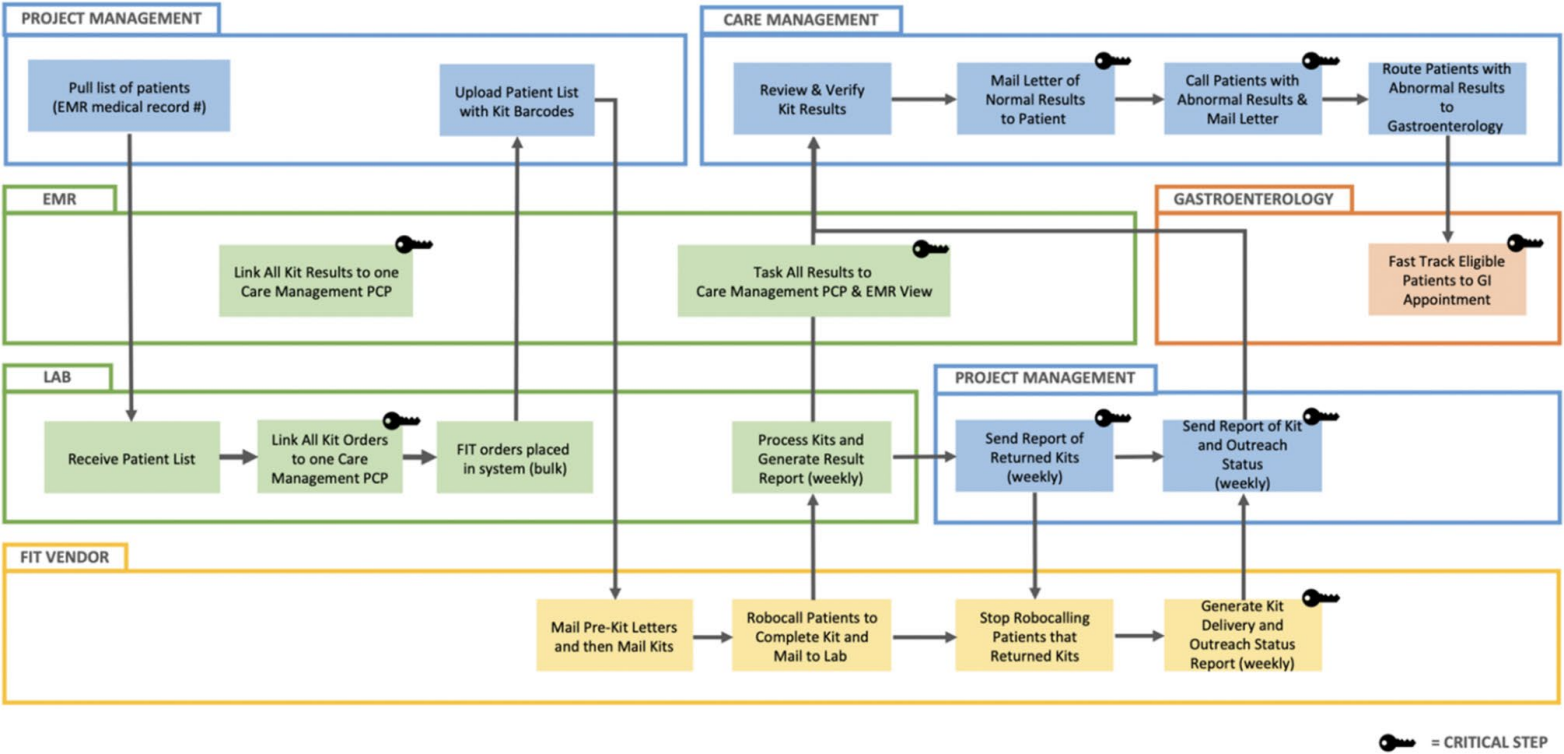
FIT at scale workflow. Multiple stakeholders (Project Management, Lab, Vendor, Care Management, Gastroenterology) identified critical steps from collaboration, feedback, and prior experience. The workflow illustrates the key elements required for each sequence to occur.

As specimens were received by the Lab team, results were populated to patients’ EHR. The clinical team members of the care management department had access to the results, and two internal medicine physicians verified them daily. For patients with normal results, we sent a letter recommending that they continue CRC screening. For patients with abnormal results, care managers made phone calls followed by a letter, which further emphasized the importance of obtaining a diagnostic colonoscopy. For patients with abnormal results whom we could not contact, we sent a certified letter. During the phone call, patients were asked whether they had a PCP or a gastroenterologist. If the patient had PCPs, we notified the PCPs by creating a task in EHR or by faxing the results. For patients without PCPs, we offered to connect them to the health system’s PCPs. All patients with abnormal results were offered fast-track referrals to gastroenterology (GI). Patients interested in fast-track referrals were assessed for their comorbidity, medications, and gastrointestinal symptoms. A GI nurse patient navigator reviewed the form within 2 business days, and patients were scheduled either for a fast-track consultation or a colonoscopy. Patients with GI symptoms (e.g. blood in their stool), a BMI greater than 35, or active cardiovascular disease (e.g. recent stent placement for coronary artery disease) were scheduled for a GI appointment (Fig. 1). Patients who declined stated they wanted to follow up with their PCPs first about finding gastroenterologists.

We leveraged tools within the EHR to establish a data feed to the Lab team platform to create centralized CRC orders. The Lab team processed the kit (a key integration element to consider with a vendor, who will process the sample), linked to a single ordering physician from the care management team. The production environment is the “live” system with which the healthcare staff interacts and where information is available and visible to all users. Orders entered the production environment, where specimen and kit specifications, including accession and barcode numbers, patient demographics, and medical record number details, were applied to each patient and used to scan the test tubes back into the system and mapped to the patient’s chart. Together, all these elements allowed for the at-scale approach, leveraging existing resources and teams to accommodate expanded volume.

#### Outcomes

The primary outcome of interest is the number and proportion of completed FIT kits. Our secondary outcome of interest is the impact on the Centers for Medicare & Medicaid Services STAR Quality Rating for the different VBC plans.

#### Analysis

We conducted a descriptive analysis of all patients and by FIT kit completion status. We then conducted a chi-square test to examine the differences in patient characteristics associated with completing the FIT kits. We examined whether there were differences in the normal versus abnormal results among patients who completed the FIT kits. To examine the impact of the program on the STAR rating, we estimated the STAR rating, including and excluding FIT kits completed through the program for each payor. This study was conducted in accordance with the Standards for Reporting Implementation Studies (StaRI) checklist.^20^

## ETHICS

This is an evaluation of an ongoing operational quality improvement project. The institutional board review at the Feinstein Institutes for Medical Research reviewed and approved the study as exempt.

## RESULTS

The GIC team leveraged the new FIT at Scale program to input FIT orders on behalf of 3680 Medicare and Medicaid patients. As kits were mailed to patients, 3466 (94.2%) were confirmed delivered with USPS mail tracking. Four hundred sixty-five (13.4%) patients completed their kit and returned it to the health system’s lab for processing between November 2023 and April 2024. In total, 465 (13.4%) kits were completed through the at-scale process—236 (50.8%) patients completed their kit without having a successful IVR call (missing number, invalid number, busy signal, disconnected), while 229 (49.2%) completed the kit upon receiving a successful IVR call (patient picked up phone call, left message). Four hundred twenty (90.3%) had normal (negative) results, and 45 (9.7%) had abnormal (positive) results (Table 1).

**Table 1:** Patient Characteristics and Screening Completion (*N* = 3680)

**Characteristic**	**Total kits** (*n* = 3680)	**Kit delivered** (*n* = 3466)	**Completed kit** (*n* = 465)	**Completed with reminders** (*n* = 229)	**Completed without reminders** (*n* = 236)	**“Normal results (neg)”** (*n* = 420)	**“Abnormal results (pos)”** (*n* = 45)
Sex							
Male	1677 (46%)	1567 (93%)	225 (14%)	110 (49%)	115 (51%)	200 (89%)	25 (11%)
Female	2003 (54%)	1899 (95%)	240 (13%)	119 (50%)	121 (50%)	220 (92%)	20 (8%)
Age							
45–55	1108 (30%)	1039 (94%)	130 (13%)	56 (43%)	74 (57%)	121 (93%)	9 (7%)
56–66	1365 (37%)	1271 (93%)	174 (14%)	90 (52%)	84 (48%)	153 (88%)	21 (12%)
67–75	1207 (33%)	1156 (96%)	161 (14%)	83 (52%)	78 (48%)	146 (91%)	15 (9%)
Race							
Asian	210 (6%)	198 (94%)	26 (13%)	11 (42%)	15 (58%)	24 (92%)	* 2 (8%)
Black or African American	356 (10%)	332 (93%)	52 (16%)	26 (50%)	26 (50%)	44 (85%)	* 8 (15%)
White	1833 (50%)	1733 (95%)	230 (13%)	127 (55%)	103 (45%)	201 (87%)	* 29 (13%)
Other/unknown	1281 (35%)	1203 (94%)	157 (13%)	65 (41%)	92 (59%)	151 (96%)	* 6 (4%)
Ethnicity							
Hispanic or Latino	468 (13%)	442 (94%)	60 (14%)	27 (45%)	33 (55%)	56 (93%)	4 (7%)
Non-Hispanic or Latino	2225 (60%)	2102 (94%)	299 (14%)	152 (51%)	147 (49%)	263 (88%)	36 (12%)
Other/unknown	987 (27%)	922 (93%)	106 (11%)	50 (47%)	56 (53%)	101 (95%)	5 (5%)
Language							
English	2940 (80%)	2773 (94%)	* 380 (14%)	195 (51%)	185 (49%)	336 (88%)	* 44 (12%)
Spanish	249 (7%)	239 (96%)	* 42 (18%)	14 (33%)	28 (67%)	41 (98%)	* 1 (2%)
Other/unknown	491 (13%)	454 (92%)	* 43 (9%)	20 (47%)	23 (53%)	43 (100%)	* 0 (0%)
Patient relationship							
New (no visit in 18 m)	1407 (38%)	1309 (93%)	* 122 (9%)	66 (54%)	56 (46%)	115 (94%)	7 (6%)
Established (visit within 18 m)	2273 (62%)	2157 (95%)	* 343 (16%)	163 (48%)	180 (52%)	305 (89%)	38 (11%)
Payer							
Payer A—Medicare	726 (20%)	691 (95%)	102 (15%)	62 (61%)	* 40 (39%)	95 (93%)	7 (7%)
Payer B—Medicare	190 (5%)	180 (95%)	33 (18%)	9 (27%)	* 24 (73%)	32 (97%)	1 (3%)
Payer B—Medicaid	2280 (62%)	2123 (93%)	276 (13%)	126 (46%)	* 150 (54%)	248 (90%)	28 (10%)
Payer C—Medicare	156 (4%)	150 (96%)	14 (9%)	8 (57%)	* 6 (43%)	11 (79%)	3 (21%)
Payer D—Medicare	328 (9%)	322 (98%)	40 (12%)	24 (60%)	* 16 (40%)	34 (85%)	6 (15%)
Total	3680	3466 (94.2%)	465 (13.4%)	**229 (49.2%)**	**236 (50.8%)**	420 (90.3%)	45 (9.7%)

^*^p-value < 0.05 denotes that the indicated patient characteristic category is associated with the FIT process measures

Results are from May 1, 2024

Twenty-four duplicate records removed (from patients with both Payer B Medicaid and Medicare)

Interactive voice response (IVR) is an automated system that allows callers to interact with a computer operated telephone system

Reasons for no IVR: kit already completed, missing or invalid phone #, no answer/mailbox setup, busy signal, disconnected

**Figure 2:**
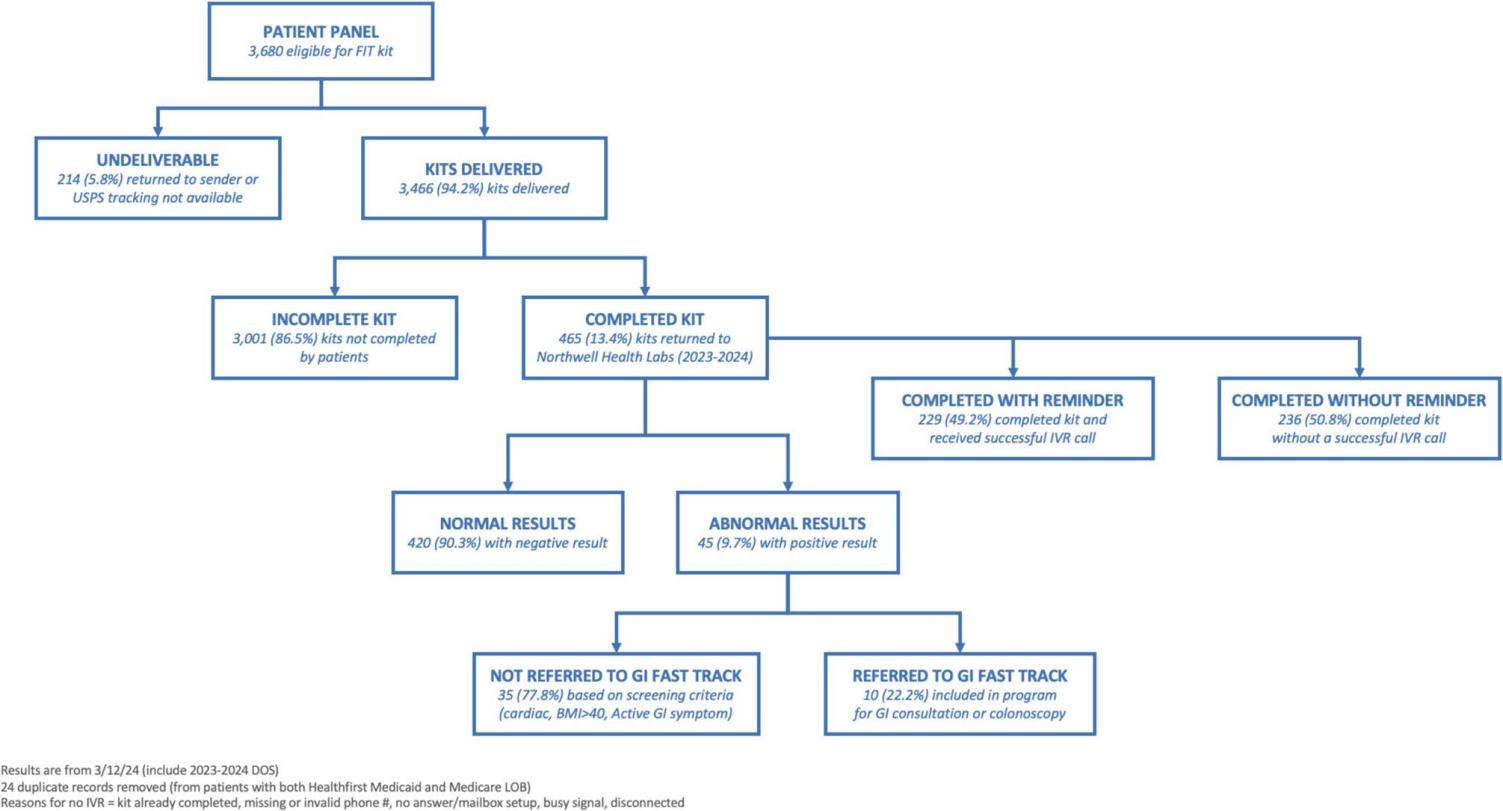
FIT at scale outcomes.

Next, we assessed whether patients with abnormal FIT results had a diagnostic colonoscopy (Table 2). Of the 45 abnormal results, 35 (77.8%) were excluded from the gastroenterology fast-track program due to cardiac or pulmonary conditions, body mass index (BMI) greater than 40, active gastrointestinal symptoms, or patient declination. Ten (22.2%) were included in the fast-track program and screened for a gastroenterology consultation and/or colonoscopy for further care (Fig. 2). As of May 2024, 11 out of 45 patients with abnormal FIT results underwent a diagnostic colonoscopy, and 10 patients were found to have tubular adenomas.

**Table 2:** Screening completion and abnormal (Positive) results (*n* = 45)

**Characteristic**	**“Abnormal results (pos)”**	**“Not referred to GI Fast Track Program”**	**“Referred to GI Fast Track Program”**	**“Colonoscopy (post kit)”**
Sex				
Male	25 (56%)	21 (84%)	4 (16%)	5 (20%)
Female	20 (44%)	14 (70%)	6 (30%)	6 (30%)
Age				
45–55	9 (20%)	6 (67%)	3 (33%)	3 (33%)
56–66	21 (47%)	18 (86%)	3 (14%)	5 (24%)
67–75	15 (33%)	11 (73%)	4 (27%)	3 (20%)
Race				
Asian	2 (4%)	2 (100%)	0 (0%)	0 (0%)
Black or African American	8 (18%)	6 (75%)	2 (25%)	3 (38%)
White	29 (64%)	22 (76%)	7 (24%)	7 (24%)
Other/unknown	6 (13%)	5 (83%)	1 (17%)	1 (17%)
Ethnicity				
Hispanic or Latino	4 (9%)	3 (75%)	1 (25%)	0 (0%)
Non-Hispanic or Latino	36 (80%)	28 (78%)	8 (22%)	10 (28%)
Other/unknown	5 (11%)	4 (80%)	1 (20%)	1 (20%)
Language				
English	44 (98%)	34 (77%)	10 (23%)	11 (25%)
Spanish	1 (2%)	1 (100%)	0 (0%)	0 (0%)
Other/unknown	0 (0%)	0 (0%)	0 (0%)	0 (0%)
Patient relationship				
New (no visit in 18 m)	7 (16%)	7 (100%)	0 (0%)	1 (14%)
Established (visit within 18 m)	38 (84%)	28 (74%)	10 (26%)	10 (26%)
Payer				
Payer A—Medicare	7 (16%)	6 (86%)	1 (14%)	2 (29%)
Payer B—Medicare	1 (2%)	1 (100%)	0 (0%)	0 (0%)
Payer B—Medicaid	28 (62%)	22 (79%)	6 (21%)	8 (29%)
Payer C—Medicare	3 (7%)	3 (100%)	0 (0%)	0 (0%)
Payer D—Medicare	6 (13%)	3 (50%)	3 (50%)	1 (17%)
Total	45	35 (77.8%)	10 (22.2%)	11 (24.4%)

Results are from 3/12/24 (include 2023–2024 date of service)

Duplicate records removed (from patients with both payer B Medicaid and Medicare)

Excluded based on screening criteria (active cardiac or pulmonary diseases, BMI > 40, active GI symptom) or patient declined

Lastly, we examined the program’s effect on estimated quality improvement and financial incentive (Table 3). Of the four payers, including Medicare Advantage and managed Medicaid programs, we saw a 1 STAR rating improvement in four instances (out of six total). From a financial perspective, the project resulted in a projected additional $1.4 million of pay for performance incentives—driven by an increase in our aggregate STAR scores on an investment of $63,000 (Table 4).

**Table 3:** Pay for performance STAR rating impact (2023)

**Payer**	**Line of business**	**All kits**	**Kits returned**	**Return rate**	**Total compliance**	**Compliance impact (2023 completion)**	**STAR rating impact (2023 completion)**	**Total measure earnings (2023 completion)**
Payer A	Medicare	726	102	14%	75.0%	1.0%	3 STAR to 4 STAR	$ 1204
Payer B								
Group 1	Medicare	43	3	7%	81.0%	1.0%	-	$ 7360
Group 2	Medicare	42	8	19%	79.0%	1.0%	-	$ 9240
Group 3	Medicaid	572	66	12%	61.0%	2.0%	-	-
Group 4	Medicare	70	18	26%	83.0%	3.0%	4 STAR to 5 STAR	$ 19,200
Group 5	Medicaid	1223	155	13%	64.0%	2.0%	2 STAR to 3 STAR	$ 56,500
Group 6	Medicare	35	4	11%	72.0%	1.0%	-	-
Group 7	Medicaid	255	25	10%	50.0%	1.0%	-	-
Group 8	Medicaid	230	30	13%	58.0%	2.0%	1 STAR to 2 STAR	-
Payer C	Medicare	156	14	9%	72.0%	0.0%	-	-
Payer D	Medicare	328	40	12%	60.0%	1.0%	-	$ 48,250
Total	-	3680	465	13%	-	-	-	$ 141,754

**Table 4:** Itemized costs

Cost category	Quantity	Price	Total cost
FIT kit mailer	3704	$ 12.00	$ 44,448.00
Pre-kit letter	3704	$ 2.50	$ 9260.00
Interactive voice response (IVR) calls	3330	$ 2.85	$ 9490.50
**Total**			**$ 63,198.50**
**Additional revenue**			
Lab fee-for-service	465	$ 10.00	$ 4650.00

## DISCUSSION

The health system–level initiative, involving multiple aligned stakeholders, improved CRC screening and ensured an end-to-end process. The advantage of this effort was its ability to perform at scale and as a transferable model through an interdisciplinary care approach. Care coordination between gastroenterology and primary care has been proven to address significant barriers in CRC screening.^21^ A registered nurse care manager to answer any concerns or questions, provide care coordinated with gastroenterologists for patients with abnormal results, and serve as a liaison between patients and the health system helped achieve these results. We were also able to incorporate and quantify VBC models as a potential means of estimating costs and return on investment for long-term sustainability. Furthermore, we were able to capture process and outcome data, including kit delivery to colonoscopy results, analyzing and quantifying the downstream effects of these interventions.

FIT screening rates and diagnostic colonoscopy completion rates among patients with abnormal results remained low, consistent with other studies.^1,22,23^ Prior studies only included patients with Medicaid and Medicare plans. A CRC screening program that included only voluntary participants resulted in markedly higher kit return rates of over 60%.^24^ However, when patients from the Medicaid program were included, utilizing reminders, the return rate (15.8%) was comparable.^25^ Patients with Medicaid may face additional barriers, suggesting that a more intensive or multimodal approach may be required to lead to higher completion rates compared to patients with private insurance. There are multiple hypotheses for the low completion rates seen in this study. First, the kits were sent during the winter holiday season, a known period where patient behavior is affected relative to the rest of the year.^26^ A subsequent quality improvement project in 2024, with FIT kit distribution in April 2024 and follow-up outreach via IVR, chat messaging, and telephone calls, showed a higher completion rate (19%). Second, the target population may require more education to complete the CRC screening. Many of these patients had primary care visits in the past year but remain unscreened for colorectal cancer. Among patients with abnormal results, the low completion rate of diagnostic colonoscopy could be multifactorial.^27–29^ We were unable to contact some patients after initial notification of abnormal results, and some patients required additional testing related to their comorbidities to receive a colonoscopy.

When creating the program, we had to consider different CRC screening products, cost of use, ease of integration, existing agreements, and health system goals. There are over 26 unique FIT kits on the market, which vary in administration and use, with differing levels of efficacy.^30^ Given those factors, we elected Polymedco FIT since we had an existing agreement, standard workflows, and the ability to have technical integration with our lab. When looking at cost, we reviewed several studies to examine this from a diverse payer perspective. One study showed an incremental cost-effectiveness ratio of $116 cost per additional person screened for a mail-based FIT program with mail-based reminders.^25^ Another study found a cost–benefit of $18 per FIT kit when combined with a phone-based reminder system.^31^ Mail-based at-home FIT programs have shown cost-effectiveness and clinical efficacy across diverse patient populations and health systems. However, only one of these studies looked at cost–benefit from a health systems perspective with Medicaid patients, and none has examined cost-effectiveness from specifically the VBC reimbursement model. The true cost and benefit of the program are hard to assess, as quantifying the time and effort of all stakeholders is complex, as well as how the improvement in the CRC screening rate contributed to the STAR rating. Therefore, the findings can be only an approximate estimate. Although the completion rate is low, we were able to complete 465 CRC screenings through this initiative, and it is possible that some patients may have completed colonoscopy after receiving the letter reminding them about the importance of getting CRC screening, which is not captured in the data. CRC screening through FIT completion has shown a lower risk of CRC death,^4^ suggesting that there is value in implementing such a program.

There are several limitations. The study population, while diverse in ethnicity and age, may have limited generalizability to the US population at large. For instance, the proportion of patients with limited English proficiency was higher than the national average. Furthermore, VBC contracts can vary highly across different states, and reimbursement models may not be easily compared. However, Medicaid and Medicare reimbursement models are more ubiquitous. For consideration, the monitored time of intervention for this study was short; kits returned as a result of intervention in 2024 were not included in the financial and performance analysis of 2023 VBC programs. Furthermore, due to the proximity to other health systems in the area, it is possible that some patients may have had CRC screenings elsewhere. For this program, we only captured FIT kits processed at our lab, but it is possible that patients may have completed CRC screenings through other modalities or at another health system.

There are opportunities to improve the completion rate. We found that established patients who were seen by their provider within the past 18 months had a significantly higher return rate than those seen beyond this time frame. Establishing a timeframe for when kits are distributed could result in higher overall return rates for at-home FIT testing. Integrating FIT as part of ambulatory visits could also hold the potential to address low return rates. Patients who are unable to be reached could be approached during their PCP’s office visit for intervention by providing education and alternative means of CRC screening.

## Supplementary Information

Below is the link to the electronic supplementary material.

ESM 1 (DOCX 879 KB)

## Data Availability

The data supporting the findings of this study are considered protected health information under HIPAA regulations and cannot be publicly shared. Researchers interested in accessing a de-identified version of the data for further analysis must submit a data use agreement and obtain approval from the Institutional Review Board. Contact the corresponding author for further inquiries regarding data access procedures.
