# Supplementary figures and images for: System-Wide Implementation of Colorectal Cancer Screening in a Value-Based Care Setting
Source: J Gen Intern Med. 2025 Jul 14;41(3):780–7. doi: 10.1007/s11606-025-09706-0 (PMC12961058; doi:10.1007/s11606-025-09706-0)

Appendix


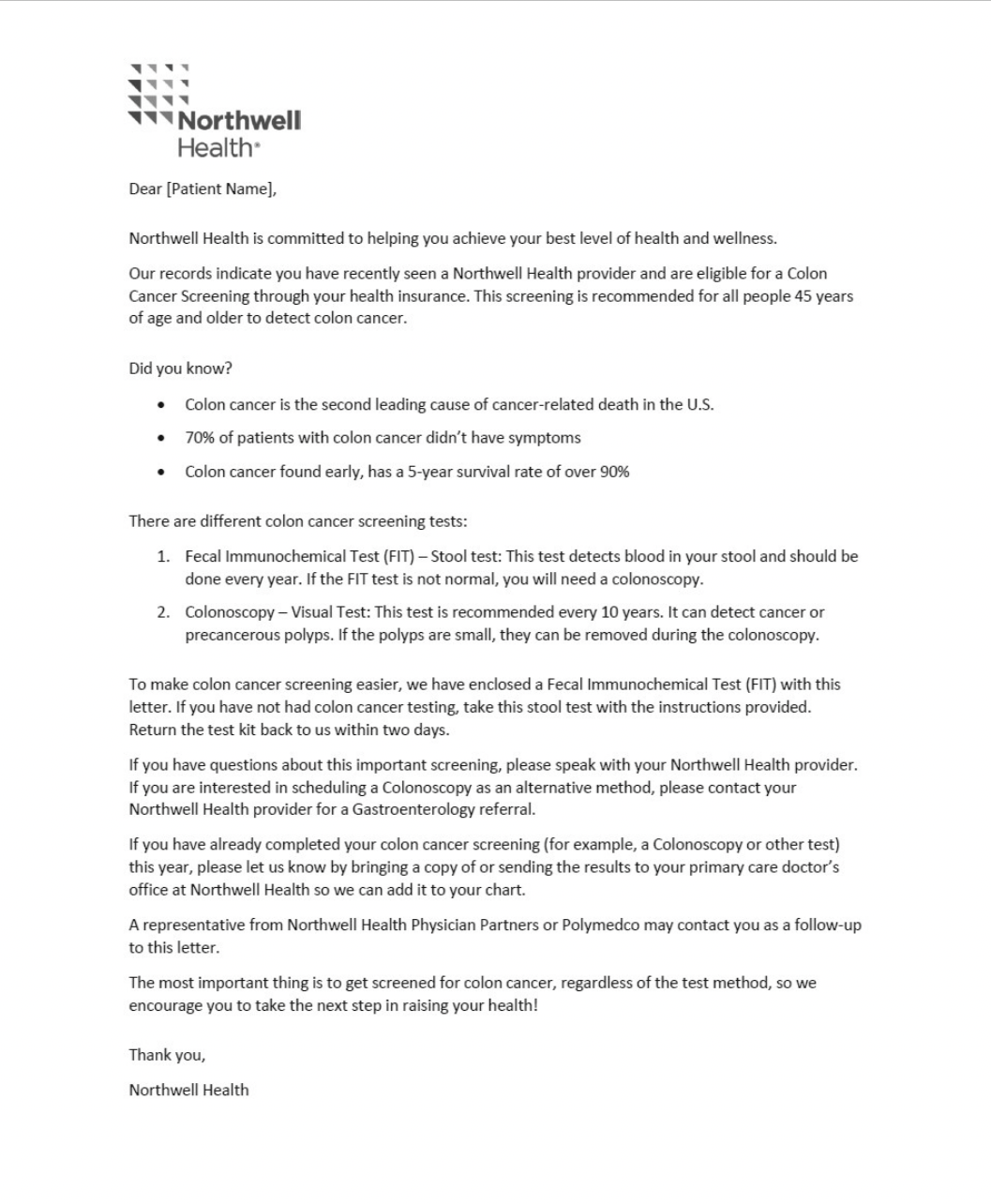

Supplement: Supplementary file 1 — (DOCX 879 KB) [file 11606_2025_9706_MOESM1_ESM.docx]
